# Pneumatic tourniquet versus no tourniquet in transfemoral amputation – a study protocol for a randomized controlled trial

**DOI:** 10.1186/s12891-022-06000-6

**Published:** 2022-12-13

**Authors:** Anna Trier Heiberg Brix, Tine Nymark, Hagen Schmal, Martin Lindberg-Larsen

**Affiliations:** 1 Department of Orthopedic Surgery and Traumatology, Odense University Hospital, Odense, Denmark; 2 Department of Clinical Research, University of Southern Denmark, Odense, Denmark; 3 Department of Orthopedics and Traumatology, University Medical Center Freiburg, Freiburg im Breisgau, Germany

**Keywords:** Major amputation, Tourniquet, Blood loss

## Abstract

**Background:**

Lower extremity amputation patients represent a frail group with extensive comorbidity. Transfemoral amputation is a high-risk procedure with 37–50% risk of mortality in the first year. Substantial blood loss during surgery increases the risk of anemic complications and death for these already weakened patients. The use of tourniquet during surgery may reduce blood loss, the need for blood transfusions, the related complications as well as the length of the surgery. However the use of tourniquet may be related to impaired wound healing and hence the use should be investigated in a randomized controlled trial.

The primary aim of this study is to investigate the total blood loss and secondary to investigate differences in complications after transfemoral amputation between patients operated with or without tourniquet.

**Methods:**

The total blood loss is calculated using Nadlers approach. Based on data from a pilot series, the sample size was calculated to 124, allocated 1:1 in two groups of 62 participants to ensure detection of at least 200 mL difference in the total blood loss. The primary outcome is the total blood loss. Secondary outcomes are blood transfusions, duration of surgery, length of hospital stay and risk of complications within 90 days (re-admissions, re-operations and mortality). Explorative outcomes are 1 year mortality and re-operation risk. Further explorative outcomes are postoperative quality of life (questionnaire EQ-5D-5L) and evaluation of number of prosthesis users including evaluation of prosthesis-specific function measured 3, 6, and 12 months postoperatively.

**Discussion:**

The possibility to enhance patient safety is highly relevant and this trial will provide data for evidence based recommendations of best practice in amputation surgery.

**Trial registration:**

The trial is registered on ClinicalTrials.gov with ID: NCT05550623. Initial release: 13/09 2022.

## Introduction

One thousand eight hundred major lower extremity amputations (LEA) are performed annually in Denmark, defined by amputation above the ankle and divided in hip disarticulation, transfemoral amputation (TFA), knee disarticulation and transtibial amputation [1, 2]. LEA procedures are common, but it is an area which lack evidence based practice [3].

Chronic leg ischemia and infection as well as the associated pain are the most common indications for non-traumatic amputations [4].

Currently it is debated whether use of tourniquet during surgery can prevent overall blood loss, reduce length of surgery and anesthesia. A tourniquet is a well-known surgical instrument and is used to control bleeding and provide a better visual overview for the surgeon. However the use of tourniquet may induce complications related to ischemia and compression of tissue such as hematoma/edema, skin necrosis, rhabdomyolysis, compartment syndrome, paresis and deep vein thrombosis [5–7].

Guidelines for tourniquet use in surgery exist, with few differences regarding duration and pressures [8]. A guideline specific for amputation surgery does not exist. Two previous studies have investigated the effect of tourniquet use in transtibial amputation surgery. Both found that tourniquet use significantly reduced the intraoperative blood loss and the need for blood transfusion postoperatively [9, 10]. Another study found tourniquet use in transtibial amputation surgery to lower reoperation rate from 38% to 14% [11].

The total blood loss in TFA surgery has been estimated to be twice the intraoperative blood loss [12]. The use of tourniquet has not been investigated in TFA surgery.

The hypothesis is that the use of tourniquet in TFA surgery will reduce the total blood loss, the intraoperative blood loss and the need for blood transfusion post-operatively, without complications and thereby improve patient safety. The aim of the trial is primary to investigate total blood loss and secondary to investigate differences in complications after TFA surgery between patients operated with use of pneumatic tourniquet versus no tourniquet.

## Method

This trial is a randomized, prospective, 1:1 allocated, two-arm, superiority, single center trial. This protocol follows the international recommendations from Standard Protocol Items: Recommendations for Interventional Trials (SPIRIT) [13]. Reports from the study will follow the CONSOlidated standards of Reporting Trials (CONSORT) [14].

### Inclusion and exclusion criteria

Inclusion and exclusion criteria are listed in Table 1. Primary TFA is defined by no earlier amputation surgery at femoral level. Participants with same site earlier lower amputation (e.g. transtibial or knee disarticulation) can be included.

**Table 1:** Inclusion and exclusion criteria

Inclusion criteria	Exclusion criteria
- Speak and understand Danish and able to give informed consent - ≥18 years of age - Indication for primary transfemoral amputation	- Bilateral amputation in same procedure - Malignant disease as main cause of amputation - Not possible to place tourniquet correctly (surgeon assessment) - Acute trauma - Planned surgery with surgeon charged less than a second year resident - Already participating in this trial

### Participants and recruitment

The participants will be recruited from Orthopedic Department, Odense University Hospital (OUH). A consultant orthopedic surgeon will always state and approve the indication for TFA surgery. All patients scheduled for primary TFA surgery will be screened for eligibility to participate based on the inclusion and exclusion criteria (Table 1). If eligible the attending physician will hand out written information and consent form.

Eligible patients will receive both written and oral information before giving final consent.

### Trial flow

One hundred twenty-four participants will be included in this trial. After written consent, baseline EQ-5D-5L is completed and a pre-operative hemoglobin value (< 4 weeks) is ensured. After randomization and amputation, the stump is examined at the 3. postoperative day, where blood test also are drawn for post-operative hemoglobin value. Surveys are send out at 3 months, 6 months and 12 months.

Patients that are not included in this RCT, no matter of cause, will be offered to participate in a concurrently prospective non-interventional cohort study where the main outcome is health related quality of life before and after surgery, accessed in surveys. The same baseline data will be recorded on these patients and they will receive standard treatment without tourniquet. This will serve as drop-out analysis.

Screening log is performed on patients not included, to examine the cause of non-inclusion.

**Fig. 1:**
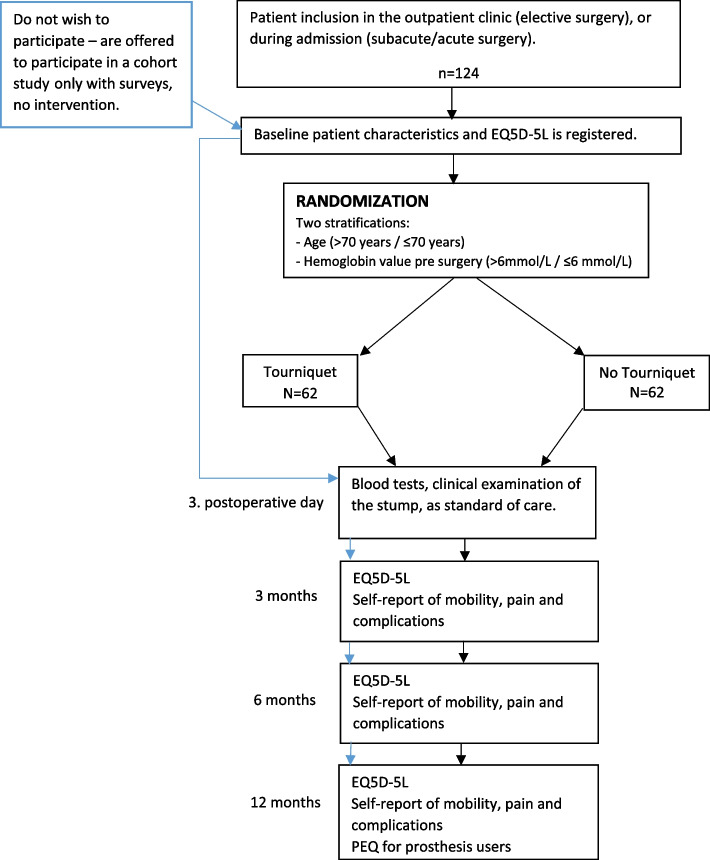
Flowchart of the trial flow. Blue arrow: Concurrent cohorte. Black arrow: RCT-trial

The flow of the study is seen in Fig. 1.

### Intervention

#### Group 1: randomized to procedure with tourniquet application

##### Sterile wash to groin and placement of sterile tourniquet.

The amputation level approximately 10–15 cm above the upper edge of patella is marked and the anterior and posterior flaps are measured and drawn out.

##### The leg is lifted, and the tourniquet is inflated. The pressure is set to 250 mmHg. Starting time is noted.

Incision through skin, fascia and musculature. The femoral vessels are clamped, cut and ligated. With an oscillating saw the femoral bone is cut, and the leg can be removed.

##### The tourniquet is deflated. Tourniquet time is noted.

The edge of the femoral bone is rasped smooth. A myodesis is performed, attaching the adductor muscle to the end of the femoral bone. Nervus Ischadicus is dissected as proximal as possible to avoid development of neuroma. Ligation of bleeding vessels. Fascia and skin is closed with sutures. A soft compression bandage is applied to the stump.

Weight of surgical swabs is noted, to estimate intraoperative blood loss.

#### Group 2: randomized to procedure without tourniquet

Same procedure as described above, but without tourniquet application.

### Postoperatively (both groups)

Hemoglobin value is measured on the third postoperative day.

Postoperatively all participants are evaluated by a multidisciplinary team, senior consultant, nurses, specialized physiotherapist and occupational therapist whether or not they are eligible for prosthesis immediately, will benefit from a structured rehabilitation period before a second evaluation, or not eligible for prosthesis (terminally ill, multimorbidity, bilateral amputees, i.e.).

The stump is examined on the third postoperative day. Discharge from admission is typically after 5–7 days. Sutures will be removed by the participants’ general practitioner or homecare nurse after 21 days. Based on the status at discharge an outpatient visit can be arranged.

The participants will receive EQ-5D-5L and a questionnaires about their daily life, mobility, and pain at 3 months, 6 months and 12 months post-operatively. Participants using a prosthesis within 12 months postoperatively will receive the Prosthesis evaluation questionnaire (PEQ) at 12 months.

### Outcomes

Overview of outcomes are listed in Table 2.

**Table 2:** Study outcomes overview

Primary	Secondary	Explorative
Total blood loss calculated using Nadler’s approach	Blood transfusions Intraoperative blood loss Length of stay Complications during admission Risk of mortality ≤30 days Risk of readmission ≤90 days Risk of re-operation ≤30 days, ≤90 days	Risk of re-operation ≤1 year Risk of mortality ≤1 year Prosthesis user ≤1 year Quality of Life (EQ-D5) Prosthesis evaluation questionnaire (PEQ)

#### Primary outcome

**Fig. 2:**
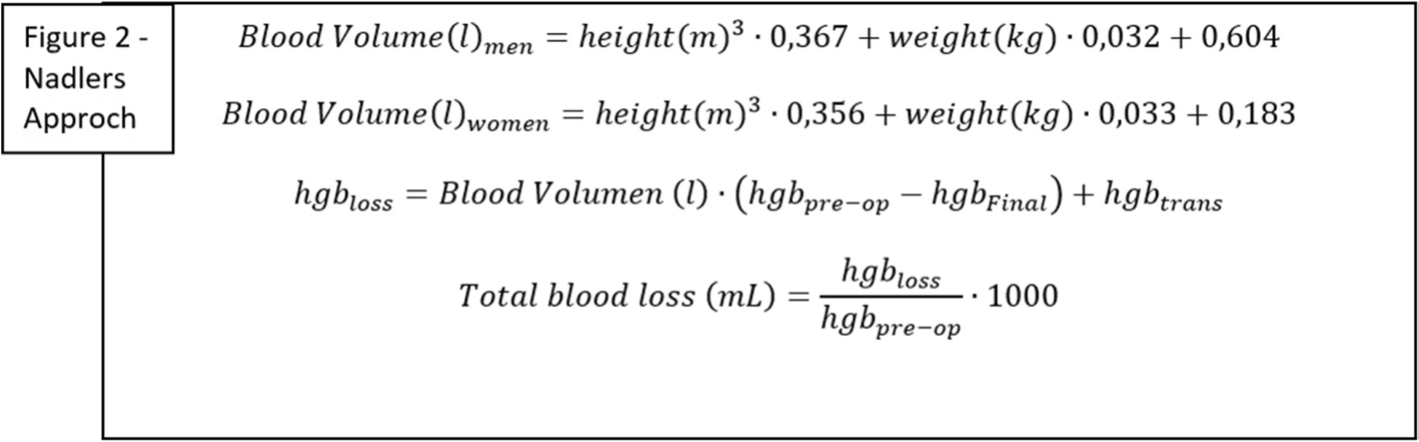
Where hgb_pre-op_ (g/L) is the hemoglobin measured before surgery (period 4 week to day of surgery), and hgb_final_ (g/L) is the final hemoglobin measured on the third postoperative day. Hbg_trans_ is the amount of hemoglobin (in grams) in the blood transfusions given before hgb_final_ (g/L) is measured. In Denmark, hemoglobin values are measured in mmol/L as standard. To convert to g/L the value is multiplied with 16.1. The amount of hemoglobin in one blood transfusion is estimated to 55 g per portion

**Total blood loss** calculated via Nadler’s Approach (Fig. 2). To calculate this outcome the following information is needed: gender, weight, height, hemoglobin value before surgery (accepted timespan 4 weeks before surgery to day of surgery), hemoglobin value on the third day post surgery, and the number of blood transfusions (from day of surgery to third postoperative day) [15].

#### Secondary outcomes

**Postoperative blood transfusions**: Transfusions received postoperatively during primary admission and potential readmissions.

**Intraoperative blood loss**: Estimated value. Measured in surgery as weight difference in surgical swabs and measured suction blood.

**Length of stay**: Length of stay is defined as postoperative nights in hospital, including transfers to other departments/hospitals, until discharge to home or rehabilitation unit.

**Complications during admission**: Complications will be grouped using the OrthoSAVES guidelines [16, 17].

**Risk of mortality ≤30 days**: Data from patient records, combined with data from the CPR-registry [18].

**Risk of readmission ≤90 days**: Readmission is defined in at least one overnight stay. Readmissions due to planned procedures or obviously unrelated to surgery are not included.

**Risk of re-operation ≤30 days and ≤ 90 days**: Defined as same site surgery, whether amputation at a higher level where the femoral bone shortened or revision of soft-tissue

#### Explorative outcomes

**Risk of re-operation ≤1 year**: Defined as same site surgery, whether amputation at a higher level where the femoral bone shortened or revision of soft-tissue. All re-operations in the one-year period after index surgery will be included for analysis.

**Risk of mortality ≤1 year**: Data from patient records, combined with data from the CPR-registry [18].

**Prosthesis user ≤1 year**: The amount of participants that will be prosthesis users in the first year after amputation.

**Quality of life (EQ-D5-5L)**: The ED-5Q-5L is a validated survey for measuring health related quality of life it consists of five dimensions: mobility, self-care, usual activities, pain/discomfort and anxiety/depression, and a visual analog scale [19]. The participants will answer EQ-5D-5L before surgery (at booking or at admission to department), 3 months after, 6 months after and 12 months after index TFA surgery.

**Prosthesis evaluation questionnaire (PEQ)**: The PEQ is translated to Danish and assesses prosthesis related quality of life in nine subscales. The Danish validation showed reliability for six subscales and mixed results for the last three subscales [20]. To use this survey prosthesis must be used. According to the Swedish amputation registry mean time from amputation to prosthesis fitting is approx. Four to five months for TFA and 2–3 months after transtibial amputation [21]. PEQ will be answered at 12 months post-surgery, for those participants that received prosthesis in the study period.

### Feasibility and timeframe

In the pilot study, feasibility of tourniquet application was assessed. The pilot-study was carried out in the department in May–July 2021, with 11 patients included.

Placement of tourniquet for TFA surgery is generally possible in > 90%. The blood tests are not always drawn at third day post-surgery, but this will be assessed in the new department algorithm for postoperative care for major lower extremity amputation patients, to ensure correct data collection in this study. The mean application time of the tourniquet was 12.15 minutes (5 minutes − 21 minutes). The timeframe for the trial is estimated for two years. The yearly flow of patients for TFA in department is approximately 100. With a sample size of 124, it is estimated that it is realistic to complete inclusion in two years.

### Sample size

 In the pilot series the total blood loss was calculated. A mean of 429 ml and standard deviation (SD) of 199 ml in 11 intervention procedures (with tourniquet) and a mean of 730 ml and SD of 446 ml in 12 control procedures (without tourniquet) was observed. The calculated blood loss in each group was normally distributed according to quantile-quantile plots. A mean difference of 200 ml was assumed to be lower than any reasonable clinically important difference, and hence this was chosen as the difference for the sample size calculation. From this, the need of 49 participants in each group for a two-sample t-test for a superiority trial was calculated. To take into account for up to 20% drop-out (e.g. due to invalid data/protocol violation/intraoperative mortality/participant wish/investigator indication), it was decided to include 62 participants in each group (49/0,8 = 61,25).

The sample size calculation was performed in Stata/IC 16.1with help from OPEN Statistics, OUH.

### Randomizing

Randomization is performed internet-based using REDCap Randomize, allocation 1:1. The randomization itself takes place in the period 4 weeks prior to surgery to immediately before surgery. The randomization is performed as a block randomization in blocks of 2, 4 and 6. It will be stratified for age (> 70 years / ≤70 years) and hemoglobin value pre surgery (> 6 mmol/L / ≤6 mmol/L). Two stratifies is acceptable for the calculated sample size.

### Monitoring

The study group monitors safety of the trial on an ongoing basis. If the number of re-operations and 30 days mortality in one group becomes twice as high as in the other group, the trial is discontinued. This applies after inclusion of minimum half of the sample size. In this case, it will be reported as a “serious adverse event” to the National Committee on Health Research Ethics and the Danish Agency for Patient Safety.

### Blood loss calculation

Nadlers approach [15] is a method to calculate the total blood loss (hidden and measured blood loss) after surgery, and have been used previously to calculate total blood loss following amputation [10, 12], as well as other orthopedic procedures [22, 23].

Nadler’s approach consists of an estimation of blood volume based on weight, height and gender and calculation of total blood loss based on estimated blood volume, combined with hemoglobin level before surgery and on the third postoperative day (Fig. 2). If blood transfusion is received after surgery, this will be added.

### Statistical analysis plan

The investigators will report descriptive statistics of patient characteristic as mean and standard deviation (SD) for normally distributed data, median and interquartile range (IQR) for non-normally distributed data, and counts and proportions for categorical characteristics.

A two-sample t-test with unequal variance will be used to compare the primary outcome (calculated blood loss). Mean and SD for each group as well as the mean difference with a 95% normal confidence interval (CI) will be reported.

The dichotomous secondary and exploratory outcomes are reported as counts and proportions, and compared with a chi-squared test (or Fisher’s exact test, if any counts are below five). Odds ratios are reported with 95% CI from crude logistic regression.

The numerical secondary and exploratory outcomes are reported either as means and SD and compared by two-sample t-test, if deemed normally distributed by quantile-quantile plots, or otherwise, as medians and IQR and compared by Wilcoxon rank-sum test if not deemed normally distributed.

All analyses will be carried out as a superiority design and as intention to treat analysis is performed for all included participants, and *p*-values below 0.05 will be considered statistically significant. Multiple imputation will be used in handling of missing data.

## Discussion

This trial investigates the use of tourniquet in TFA surgery. A method that has been used in many years, also in Denmark, but with no evidence of providing a better outcome for the patient. If tourniquet use is proven to reduce blood loss without impaired outcome afterwards, this method should be promoted.

The frequency in use of tourniquet among surgeons does also have a cultural aspect, which differ from country to country, department to department and within the felt of orthopedics.

It might be a challenge to get long-term patient reported outcomes on this frail and weakened patient group, with a 37–50% risk of mortality in the first year [24]. This means that those who will be able to answer at 12 months not necessary are representative for the patient group. The patient group does have diversity to it, even though those with acute trauma and amputation due to cancer is excluded. The indication for amputation, besides chronic ischemia, pain and infection, might also be failure of knee arthroplasty or pain/poor function due to earlier trauma. These patients are typically younger and has a different pattern of co-morbidity than the most frequent TFA patient. The study is not limited to the atherosclerotic patients.

To our knowledge this is the first study protocol of a randomized controlled trial that investigate the use of tourniquet in TFA surgery. The data from this study makes it possible to evaluate the use of tourniquet in amputation surgery. Because of the concurrent cohort study (drop-out analysis), the representativeness of patients included can be evaluated.

### Trial status

The first participant was included the 10/102022. Status of the trial the 1/12 2022 is fifteen patients included.

## Data Availability

The datasets used and/or analyzed during the current study are available from the corresponding author on reasonable request.

## Abbreviations

LEA: Lower extremity amputation
TFA: Transfemural amputation
OUH: Odense University Hospital
PEQ: Prothesis Evaluation Questionnaire
SD: Standard deviation
IQR: interquartile range
CI: Confidence interval
